# Deficits in proactive avoidance and neural responses to drinking motives in problem drinkers

**DOI:** 10.21203/rs.3.rs-3924584/v1

**Published:** 2024-02-09

**Authors:** Thang Le, Takeyuki Oba, Luke Couch, Lauren McInerney, Chiang-Shan Li

**Affiliations:** Yale University; Yale University; Yale University; Yale University; Yale University

## Abstract

Physical pain and negative emotions represent two distinct drinking motives that contribute to harmful alcohol use. Proactive avoidance which can reduce problem drinking in response to these motives appears to be impaired in problem drinkers. However, proactive avoidance and its underlying neural deficits have not been assessed experimentally. How these deficits inter-relate with drinking motives to influence alcohol use also remains unclear. The current study leveraged neuroimaging data collected in forty-one problem and forty-one social drinkers who performed a probabilistic learning go/nogo task that involved proactive avoidance of painful outcomes. We characterized the regional brain responses to proactive avoidance and identified the neural correlates of drinking to avoid physical pain and negative emotions. Behavioral results confirmed problem drinkers’ proactive avoidance deficits in learning rate and performance accuracy, both which were associated with greater alcohol use. Imaging findings in problem drinkers showed that negative emotions as a drinking motive predicted attenuated right insula activation during proactive avoidance. In contrast, physical pain motive predicted reduced right putamen response. These regions’ activations as well as functional connectivity with the somatomotor cortex also demonstrated a negative relationship with drinking severity and positive relationship with proactive avoidance performance. Path modeling further delineated the pathways through which physical pain and negative emotions, along with alcohol use severity, influenced the neural and behavioral measures of proactive avoidance. Taken together, the current findings provide experimental evidence for proactive avoidance deficits in problem drinkers and establish the link between their neural underpinnings and alcohol misuse.

## Introduction

Drinkers may maladaptively engage in excessive alcohol use to alleviate aversive states including physical pain and negative emotions ^1,2^. Conversely, individuals can reduce harmful drinking by employing proactive avoidance, or the initiation of overt behaviors to prevent or address negative situations ^3–5^. Proactive avoidance has been found to help one refrain from drinking during alcohol cue exposure and craving ^6,7^. In problem drinkers, proactive avoidance appears to be impaired, as suggested in studies examining behavioral coping strategies including support seeking, problem solving, and goal setting ^8–10^. Despite the potentially crucial role of proactive avoidance in the prevention and reduction of harmful alcohol use, most investigations did not operationalize proactive avoidance, relying instead on questionnaires and self-reports of drinkers’ coping methods. Thus, experimental modeling is needed to better understand how proactive avoidance may be engaged to mitigate physical pain and/or negative emotions and temper the motivation to drink.

Individuals harbor distinct motives to drink. Due to ethanol’s analgesic properties, some seek alcohol to manage physical pain, which contributes to the development and maintenance of problem drinking. Indeed, problem relative to social drinkers reported greater physical pain as well as use of alcohol to cope ^11,12^. In individuals receiving treatment for alcohol use disorders (AUD), physical pain level at the end of treatment was predictive of drinking frequency 12 months post-treatment ^13^. AUD patients who experienced pain reduction during treatment demonstrated a significantly lower risk of relapse compared to those who did not ^14^. Similarly, negative emotions have been associated with heavier alcohol use in drinkers who seek alcohol to cope ^15–18^. From the modeling of different drinking motives, drinking to regulate emotional distress was found to be one of the most significant predictors of alcohol consumption ^19^. As the sensory and affective dimensions of pain involve both shared and distinct circuits ^20^, it would be of interest to distinguish the brain processes underlying proactive avoidance of physical pain vs. negative emotions and elucidate how they modulate drinking behavior.

While the neural underpinnings of the interaction between emotion and action remain inconclusive, several studies have posited the role of the limbic motor circuits ^21–23^, providing a potential link between dysfunctional proactive avoidance and problem drinking. In response to physical pain, brain regions such as the insula, precentral gyrus, anterior/mid cingulate cortex, and putamen show heightened activity ^24,25^. Notably, both the insula and putamen are part of the limbic motor circuits that not only respond to aversive states but also modulate the motor processes to support proactive avoidance. For instance, the insula and striatum, including both caudate and putamen, were significantly activated during learning to avoid unpleasant events such as monetary loss and social stress ^26–29^. These regions’ activities have further been associated with avoidance behaviors during exposure to potential threats or harms ^30,31^. Importantly, an extensive literature has demonstrated insular and striatal circuit dysfunctions in problem drinkers ^32–34^. Thus, a critical question is whether problem drinking is associated with deficits in proactive avoidance along with insular and striatal circuit dysfunctions and how these deficits may interact with motives to modulate alcohol use.

The present study investigated the neural processes underlying proactive avoidance in relation to the drinking motives of physical pain and negative emotions. We employed a probabilistic learning go/nogo task (PLGT) to operationalize proactive avoidance. To determine whether problem drinkers exhibited proactive avoidance deficits, we examined behavioral performance as well as constructed reinforcement learning models to further assess learning indices. Next, we characterized brain activities and connectivities underlying proactive avoidance deficits in problem drinkers. Specifically, using whole-brain regressions, we triangulated the shared neural correlates of proactive avoidance, drinking severity, and drinking motives associated with physical pain and negative emotions. We tested the hypothesis that the putamen and insula would be involved in reflecting individual differences in these drinking motives. Finally, path models were constructed to delineate the distinct neuropsychological pathways in which physical pain and negative emotions may differentially motivate alcohol use via proactive avoidance deficits.

## Methods

### Participants

Forty-one problem drinkers (8 females, 38.8 ± 10.7 years in age) and 41 social drinkers (19 females, 35.7 ± 11.2 years) were recruited from New Haven and surrounding areas via advertisement. Participants were screened to exclude major medical, neurological, and Axis I psychiatric disorders. Exclusion criteria also included MR contraindications and history of seizures, traumatic brain injury or concussions. No participants were currently on psychotropic medications, and all tested negative for illicit substances and alcohol use via a drug and breathalyzer test, respectively, on the day of the study. Subjects provided written informed consent after details of the study were explained, in accordance with the institute guidelines and procedures approved by the Yale Human Investigation Committee.

### Assessment and behavioral task

Participants completed detailed alcohol use assessments including recent drinking history (frequency, quantity, motivation, and drinking contexts) as well as the Alcohol Use Disorders Identification Test (AUDIT) ^35^. AUDIT scores are calculated from the sum of 10 self-report questions: 3 on quantity of alcohol use, 4 on alcohol-related problems and adverse reactions, and 3 on drinking behavior. Each question receives a score from 0 to 4. A higher score indicates greater risk for having or developing an AUD. Based on the assessment of alcohol use, participants were assigned to the problem drinker group if they reported (1) an AUDIT score of 8 or higher (see *Supplementary Methods* and *Fig. S1* for additional data and rationale), and (2) weekly consumption of at least 14 drinks for men and 7 drinks for women, consistent with previous reports ^36–39^. Participants also completed the 42-item version of the Inventory of Drinking Situations (IDS-42) ^40^. The IDS-42 assesses relative frequency of drinking behavior across eight categories of drinking situations and determines the drinking motives and circumstances leading to excessive alcohol use in problem drinkers ^41,42^. The 8 drinking situation subscales of the IDS-42 are Negative Emotions, Physical Discomfort, Conflict with Others, Testing Personal Control, Urges and Temptations, Social Pleasure, Pleasant Times with Others, and Pleasant Emotions. We focused on Negative Emotions and Physical Discomfort subscales, which have been previously identified as the two major drinking motives for problem drinkers ^10,43^. Participants’ demographics and alcohol use data are detailed in *Supplementary Table S1*. To control for potential confounding effects of sex, age, education, and smoking status, we included these variables in all regression analyses as covariates.

### Probabilistic learning go/no-go task (PLGT)

**Figure 1:**
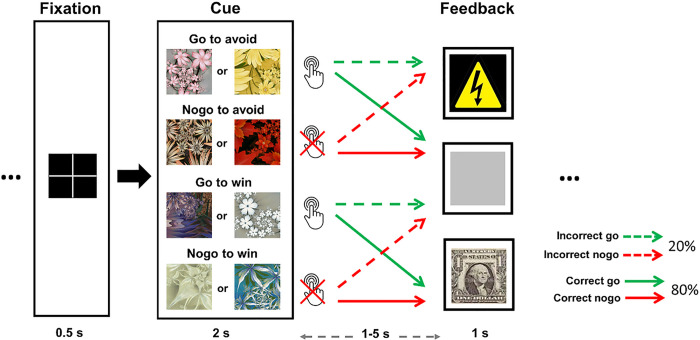
Probabilistic learning go/nogo task (PLGT): Participants learned to respond to four cue categories to avoid electric shocks (i.e., go-to-avoid, nogo-to-avoid) and gain monetary rewards (i.e., go-to-win, nogo-to-win), with two different images per cue category. Correct responses yielded favorable outcomes 80% of the times whereas incorrect responses yielded unfavorable outcomes 80% of the times. Shocks were only delivered in 50% of the shock feedback instances to reduce head movement.

Participants underwent fMRI while performing the PLGT (Fig. 1) ^44,45^. In each run, a cue (fractal) image was presented at the beginning of a trial to signal one of the four contingencies: go to win $1, nogo to win $1, go to avoid a painful shock, or nogo to avoid shock. There were 8 images, 2 per cue category, with cue-outcome mappings randomized across participants. The cue was displayed for 2 s and participants were instructed to decide whether to press a button (go) or not (nogo) before it disappeared. After a randomized interval of 1 to 5 s, feedback of reward (win trials), shock (avoid trials), or “null” (both win and avoid trials) was delivered. The inter-trial interval (ITI) varied randomly from 2 to 8 s. The randomization and intervening time intervals enabled modeling of distinct regional responses to anticipation and feedback. The outcome was probabilistic, with 80%/20% of correct/incorrect responses in the win trials rewarded and the remaining 20%/80% of correct/incorrect responses leading to a null outcome. In avoid trials, electric shocks were avoided (a null outcome) on 80%/20% of correct/incorrect responses, with the remainder leading to electric shocks. In reality, despite the feedback display of the shock image, shocks were randomly delivered only half of the times to minimize head movements. Each shock was followed by a 20-s rest window to allow neural and physiological responses to return to baseline.

With four 10-minute learning runs, there were approximately 50 trials for each cue. Participants won ~$72 on average (plus a base payment of $25) and experienced a total of 15 actual shocks and 15 omitted shocks on average. Prior to MRI, the appropriate pain intensity level was calibrated for each participant so that the shocks would be painful but tolerable (*Supplemental Methods*). Participants also practiced on a version of the task with identical rules. To ensure the subjects’ efficient task comprehension, there was one image per cue category in the practice version.

### Reinforcement learning (RL) models

We constructed RL models of the problem and social drinkers’ behavioral data (*Supplemental Methods*). A detailed description of the models can be found in our previous work ^45^ and elsewhere ^44^. Briefly, all models assigned an action value to each action in a given trial. Action value was updated based on the learning rate. We included the subjective impact of outcomes, which was a free parameter representing the effect size of reinforcement for a subject. We also included two other parameters validated in prior studies to better explain behavioral performance ^44,46^, namely the action bias, a tendency to press a button regardless of learning, and the Pavlovian factor, which expresses the effect of a stimulus value independent of learning.

The outputs of the models included the learning rate, which can be separated according to the sign of prediction error (PE) (positive or negative). Models that produce the learning rates for the signed PE allow an asymmetric effects of better or worse (than expected) outcome on learning ^47^. Furthermore, the learning rates can be modeled separately for each trial type. Here, we focused on the Go-to-avoid trials. The subjective impact of outcomes could also differ between win and avoid trials. In sum, we examined a total of 12 parameters and identified the best combination of these parameters in modeling the behavioral data.

Free parameters were estimated for each participant via a hierarchical type II maximum likelihood procedure, as with previous studies ^44,48^. To perform the estimation, the likelihood was maximized by the expectation-maximization procedure using the Laplace approximation to calculate the posterior probability. We used the Rsolnp package in R to optimize the likelihood functions. These models were evaluated with the integrated Bayesian information criterion (iBIC). The iBIC values approximated the log marginal likelihoods with a penalty for the number of free parameters. A smaller iBIC value represents a better model.

### Imaging protocol and data preprocessing

Conventional T1-weighted spin echo sagittal anatomical images were acquired for slice localization using a 3T scanner (Siemens Trio). Anatomical images of the functional slice locations were next obtained with spin echo imaging in the axial plane parallel to the AC–PC line with (TR) = 1900 ms, echo time (TE) = 2.52 ms, bandwidth = 170 Hz/pixel, FOV = 250 × 250 mm, matrix = 256 × 256, 176 slices with slice thickness = 1 mm and no gap. Functional blood oxygenation level-dependent (BOLD) signals were acquired using multiband imaging (multiband acceleration factor = 3) with a single-shot gradient echo echoplanar imaging sequence. Fifty-one axial slices parallel to the AC–PC line covering the whole brain were acquired with TR = 1,000ms, TE = 30ms, bandwidth = 2,290 Hz/pixel, flip angle = 62°, field of view = 210 × 210mm, matrix = 84 × 84, with slice thickness = 2.5mm and no gap.

Imaging data were preprocessed using SPM12 (Wellcome Trust Centre for Neuroimaging). Subjects with BOLD runs with significant motion (> 3-mm translation peak-to-peak movement and/or 1.5-degree rotation) were removed. Furthermore, we calculated framewise displacement (FD) for each task run and removed subjects with averaged FD of greater than 0.2. This resulted in the removal of 2 social drinkers and 4 problem drinkers, leaving a sample of 82 subjects as reported above. Images from the first five TRs at the beginning of each run were discarded to ensure only BOLD signals at steady-state equilibrium between RF pulsing and relaxation were included in analyses. Physiological signals including respiration and heart rate were regressed out to minimize the influence of these sources of noise. Images of each subject were first realigned (motion corrected) and corrected for slice timing. A mean functional image volume was constructed for each subject per run from the realigned image volumes. These mean images were co-registered with the high-resolution structural image and then segmented for normalization with affine registration followed by nonlinear transformation. The normalization parameters determined for the structure volume were then applied to the corresponding functional image volumes for each subject. Images were resampled to 2.5 mm isotropic voxel size. Finally, the images were smoothed with a Gaussian kernel of 4-mm FWHM.

### Imaging data modeling and group analyses

We constructed a general linear model (GLM) to examine the brain processes underlying the initiation and inhibition of an action to avoid painful shocks. To this end, we focused on two trial types, Go-to-avoid and Nogo-to-avoid, with cue onsets of individual trials convolved with a canonical HRF and with the temporal derivative of the canonical HRF and entered as regressors in the GLM (Friston et al., 1995). We used the contrast Go-to-avoid > Nogo-to-avoid (proactive avoidance, hereafter) to identify regional activities during proactive avoidance for individual subjects. Serial autocorrelation caused by aliased cardiovascular and respiratory effects were corrected by the FAST model.

To identify brain regions involved in proactive avoidance, we used the contrast Go-to-avoid > Nogo-to-avoid in social drinkers and used these regions in a functional connectivity analysis in problem drinkers (See Functional connectivity analysis). We additionally determined group differences of the neural processes underlying proactive avoidance at the whole-brain level. Specifically, we conducted a two-samples t-test comparing social vs. problem drinkers, using the contrast Go-to-avoid > Nogo-to-avoid as well as Go-to-avoid > 0.

To identify the neural correlates of individual differences in drinking severity and drinking motives in problem drinkers, we used whole-brain multiple regressions with each of the AUDIT, Negative Emotions, and Physical Discomfort scores as the predictor of the brain activity of proactive avoidance during the cue period. Note that imaging data from the social drinkers were not included in these multiple regressions. For exploratory purposes, we combined both subject groups and performed the same analyses and found similar results.

The results of all whole-brain analyses were evaluated with voxel p < 0.001 in combination with cluster p < 0.05, corrected for family-wise error of multiple comparisons, according to current reporting standards ^49,50^. All peaks of activation were reported in MNI coordinates.

### Functional connectivity analysis

The functional connectivity of the insula and putamen during proactive avoidance was estimated using the psychophysiological interactions (PPI) via the gPPI toolbox ^51^. A PPI model was created for each subject with three components: the physiological term which represents the time series from the seed region, the psychological term which represents the task conditions (i.e., Go-to-avoid, Nogo-to-avoid), and the psychophysiological interaction term. The PPI was computed as the element-by-element product of the deconvolved time series of the seed region and a task condition vector. We used the right insula and putamen as the seed regions which were independently defined from the multiple regression analysis (see Results). The two cue conditions were included in the model. The PPI of proactive avoidance condition was calculated by contrasting the Go-to-avoid and Nogo-to-avoid conditions.

To characterize insula and putamen connectivities during proactive avoidance, we used data from the social drinkers and employed the contrast Go-to-avoid > Nogo-to-avoid to first identify the brain regions involved in proactive avoidance. From these results, we specifically focused on the left somatomotor cortex, including the pre/postcentral gyri, due to its role in motor control, and previously reported evidence of its connectivity with the affective circuits in regulating alcohol use ^52^ as well as involvement in learning of avoidance behavior ^53,54^ (see Results). In a region-of-interest analysis, parameter estimates (β weight) of the PPI term between regions and the experimental condition were extracted. The parameter estimates represented the connectivity strength between two given regions and were examined in relation to drinking severity and proactive avoidance performance accuracy.

### Path analysis

We conducted path analyses to examine the relationship between drinking motives, drinking severity, and brain activities as well as performance accuracy in proactive avoidance. We tested two specific models, one involving Negative Emotions drinking motive and insular response during proactive avoidance and the other involving Physical Discomfort drinking motive and putamen response (see Results). Path analysis involves a set of exogenous variables with variance not accounted for by the model and endogenous variables with variance explained in part by other variables in the model ^55,56^. Path analysis is conducted with regression analysis which predicts the effects of all other variables on the endogenous variables. The beta weights (β) from these multiple regressions represent the path coefficients. Standardized path coefficients convey assumptions about the directionality of interactions between variables. Model fit is assessed with fit indices including the Root Mean Square Estimation of Approximation (RMSEA, ≤ 0.08 for an good fit), Chi-square (χ^2^/df, ≤ 3), Comparative Fit Index (CFI, ≥ .9), and Standardized Root Mean Square Residual (SRMR, ≤ .09) ^57,58^. Paths connecting variables are assessed for significance using p < .05 as the threshold. The analysis was performed with package Lavaan ^59^ in R (https://www.r-project.org).

## Results

### Behavioral and clinical results

**Figure 2:**
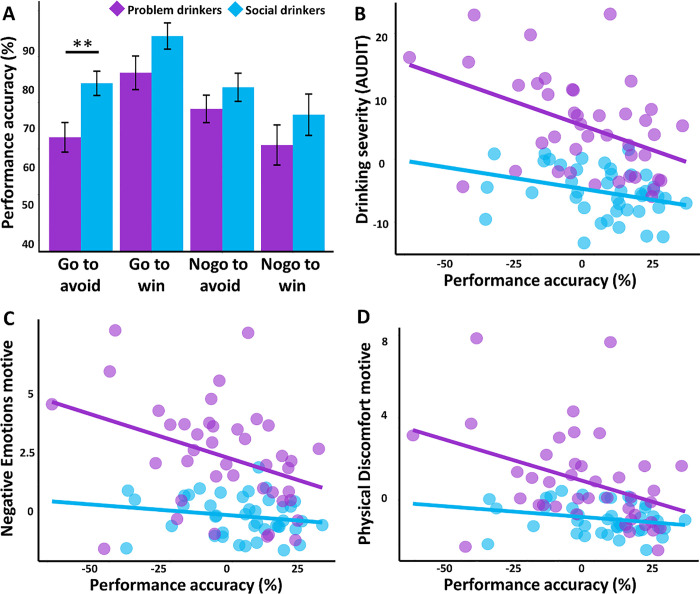
Behavioral performance and drinking characteristics. (**A**) Problem drinkers (in purple) performed significantly worse than social drinkers (in blue) in Go-to-avoid trials. Problem drinkers’ Go-to-avoid performance accuracy showed a significant negative correlation with (**B**) drinking severity as measured by AUDIT scores, (**C**) Negative emotions drinking motive, and (**D**) Physical discomfort drinking motive score. All correlation plots show regression residuals with the effects of sex, age, education, and smoking status removed. ** p < .01.

We conducted a group (problem vs. social drinkers) × motivational goal (avoid vs. win) × response type (go vs. nogo) ANOVA of performance accuracy (Fig. 2A). The results showed a significant main effect of group (*F*_*(1,320)*_ = 9.94, *p* = .001) and response type (*F*_*(1,320)*_ = 7.81, *p* = .005) but not motivational goal (*p* = .28). There was also a significant motivational goal × response type interaction effect (*F*_*(1,320)*_ = 15.04, *p* < .001). Post hoc analyses revealed that participants performed significantly better in go relative to nogo trials (*p* = .01) whereas there was no significant difference between avoid and win trials (*p* = .09). Importantly, problem drinkers performed significantly worse than social drinkers in Go-to-avoid trials (*p* = .006). The between-group differences for other three trial types (i.e., Nogo-to-avoid, Go-to-win, Nogo-to-win) were not significant (*p*’s > .08).

As expected, problem drinkers reported significantly greater drinking severity than social drinkers (*p* < .001). Relative to social drinkers, problem drinkers also reported significantly higher drinking motives of Negative Emotions (*p* < .001) and Physical Discomfort (*p* < .001). In problem drinkers, drinking severity was significantly and positively correlated with both drinking motive scores of Negative Emotions (*r* = .66, *p* < .001) and with Physical Discomfort (*r* = .68, *p* < .001). These relationships were not significant for social drinkers (*p*’s > .70).

Next, we found that problem drinkers’ Go-to-avoid accuracy was significantly and negatively correlated with drinking severity (*r* = − .44, *p* = .009, Fig. 2B), Negative Emotions (*r* = − .38, *p* = .02, Fig. 2C), and Physical Discomfort (*r* = − .43, *p* = .01, Fig. 2D) drinking motives. Social drinkers’ Go-to-avoid performance accuracy was significantly correlated with drinking severity (*r* = − .38, *p* = .02) but not the two drinking motives (*p*’s > .07).

### Reinforcement learning model results

We constructed 14 models with different combinations of free parameters to determine the model that optimally predicted the choice data (*Supplementary Fig. S2*). Using a stepwise procedure for model comparison and selection, we added one free parameter to a model, calculated the iBIC, and accepted the parameter that decreased the iBIC the most at each step. The Pavlovian factor reduced the iBIC of the basic model (one learning rate and one subjective impact of outcomes) over the other parameters. The iBIC was diminished by separation of the learning rates into positive and negative PEs and for win (i.e., monetary win) and avoid (i.e., pain avoidance) trials. The subjective impact of outcomes was separated for win and avoid trials. Finally, the action bias parameter reduced the iBIC. Thus, the optimal model included eight different learning rates (two per trial type) and two subjective impact of outcomes, action bias b, and the Pavlovian factor.

**Figure 3:**
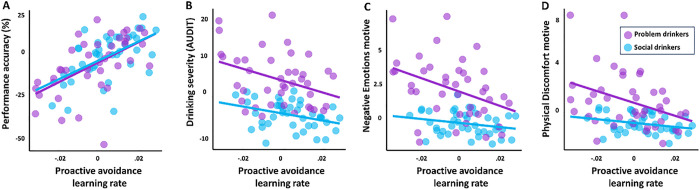
In problem drinkers, proactive avoidance learning rate showed significant correlations with (A) Go-to-avoid performance accuracy, (B) drinking severity, (C) Negative emotions, and (D) Physical discomfort drinking motives.

As problem drinkers showed evidence of proactive avoidance deficits, we focused on the learning rate. We found that their proactive avoidance learning rate with positive PE (i.e., learning when outcomes were better than expected) during Go-to-avoid trials was significantly lower than that in social drinkers (*p* = .007). Moreover, problem drinkers’ learning rate showed a significant correlation with Go-to-avoid accuracy (*r* = .58, *p* < .001), drinking severity (*r* = − .37, *p* = .022), Negative Emotions (*r* = − .36, *p* = .026), and Physical Discomfort (*r* = − .38, *p* = .020) motives (Fig. 3).

In social drinkers, learning rate with positive PE during Go-to-avoid trials was significantly correlated with performance accuracy (*r* = .52, *p* < .001) but not with the drinking severity or motive measures (*p*’s > .56). Note that all regressions controlled for sex, age, education, and smoking status.

### Imaging results

#### Whole-brain group differences

Due to the group differences in behavioral performance of proactive avoidance, we conducted a whole-brain two-samples t-test using the contrast Go-to-avoid > Nogo-to-avoid. The analysis did not result in any significant activations. Next, we examined the contrast Go-to-avoid > 0. The two-samples t-test showed greater primary visual cortex in social drinkers relative to problem drinkers (*Supplementary Fig. S3*). Problem drinkers did not exhibit any significantly greater activations relative to social drinkers.

##### Neural correlates of drinking severity.

**Figure 4:**
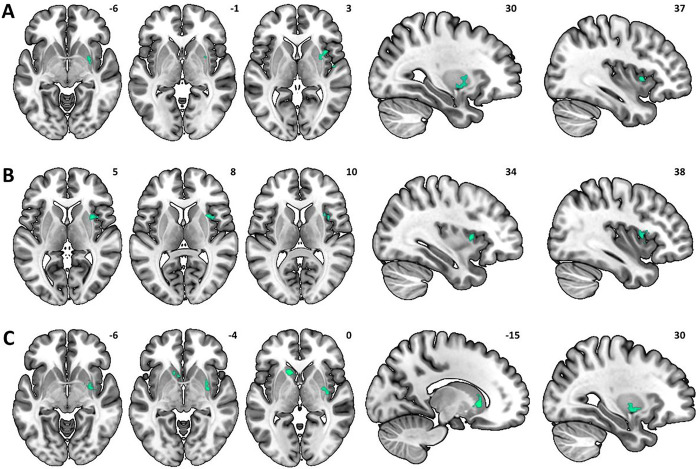
Neural correlates of drinking severity and drinking motives during proactive avoidance (Go-to-avoid > Nogo-to-avoid). (**A**) AUDIT scores predicted lower activations in the right putamen and right insula. Negative emotions and Physical discomfort drinking motive scores predicted lower activations in the right insula (**B**) and right putamen and left caudate (**C**), respectively.

Whole-brain multiple regression with the AUDIT scores as the predictor of brain activity during proactive avoidance identified the right putamen and insula in a negative correlation (Fig. 4A, *Table S2*). No clusters showed a significant positive correlation at the same threshold.

##### Neural correlates of Negative Emotion drinking motive.

Multiple regression with Negative Emotions scores as the predictor of brain activation to proactive avoidance in problem drinkers showed significantly lower activity in the right insula (Fig. 4B, *Table S2*). No clusters showed a significant positive correlation.

##### Neural correlates of Physical Discomfort drinking motive.

Multiple regression with Physical Discomfort scores as the predictor of brain activation to proactive avoidance in problem drinkers showed significantly lower activity in the right putamen and left caudate (Fig. 4C, *Table S2*). No clusters showed a significant positive correlation.

##### Shared correlates for drinking severity and motives.

From the multiple regression results, we found that the neural correlates of drinking severity and Negative Emotions drinking motive both involved the right insula. In contrast, the neural correlates of drinking severity and Physical Discomfort drinking motive both involved the right putamen. Further, Go-to-avoid accuracy showed significant and positive correlations with both the insular (*r* = .38, *p* = .016) and putamen (*r* = .47, *p* = .002) activity.

**Figure 5:**
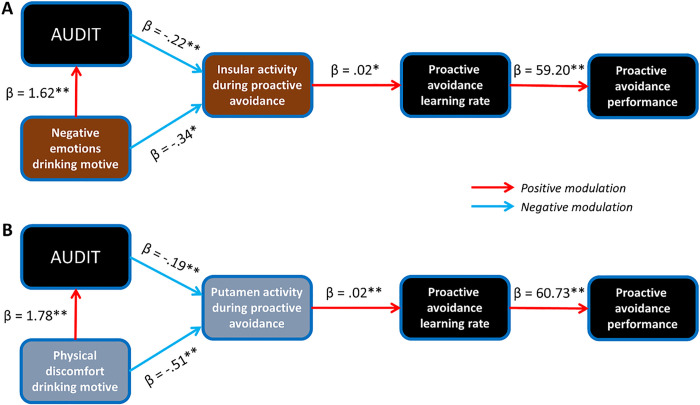
Path analysis. (**A**) Negative emotions motivated drinking, both of which reduced insular activity during proactive avoidance. Attenuated insular activity worsened behavioral performance during proactive avoidance via lowered learning rate. (**B**) Physical discomfort motivated drinking, both of which reduced putamen activity during proactive avoidance. Attenuated putamen activity worsened behavioral performance during proactive avoidance via lowered learning rate. Both models showed a good fit. * p < .05, ** p < .01

To characterize the relationships of the two drinking motives, the neural correlates of proactive avoidance, drinking severity, as well as proactive avoidance learning rate and accuracy, we constructed two separate path models. In the first, negative emotions promoted drinking, both of which then reduced insular activity during proactive avoidance (Fig. 5A). The attenuated insular activity, in turn, predicted lower proactive avoidance learning rate which influenced Go-to-avoid accuracy. The model showed a good fit (fit indices: root mean square estimation of approximation = 0.04 [90% CI: 0.00 .185], χ 2/df = 1.08, standardized root mean square residual = .09, and comparative fit index = .99). Specifically, insular activity was negatively modulated by both Negative Emotions drinking motive (*β* = − .34, *p* = .013) and drinking severity (*β* = − .22, *p* < .001). Negative Emotions drinking motive positively modulated drinking severity (*β* = 1.62, *p* < .001). Finally, insular activity positively modulated proactive avoidance learning rate (*β* = .02, *p* = .036) which, in turn, predicted Go-to-avoid accuracy (*β* = 59.20, *p* < .001).

In the second model, physical discomfort promoted drinking, both of which then reduced putamen activation to proactive avoidance (Fig. 5B). The attenuated putamen activity, in turn, predicted lower Go-to-avoid accuracy via decreased learning rate. The model showed a good fit (fit indices: root mean square estimation of approximation = 0.00 [90% CI: 0.00 0.192], χ 2/df = .79, standardized root mean square residual = .09, and comparative fit index = 1.00). Specifically, drinking severity was positively modulated by Physical Discomfort drinking motive (*β* = 1.78, *p* < .001). Putamen activity was negatively modulated both by Physical Discomfort drinking motive (*β* = − .51, *p* = .001) and drinking severity (*β* = − .19, *p* = .001). The putamen activity then positively modulated proactive avoidance learning rate (*β* = .02, *p* < .001), which predicted Go-to-avoid accuracy (*β* = 60.73, *p* < .001).

##### Functional connectivity analysis.

**Figure 6:**
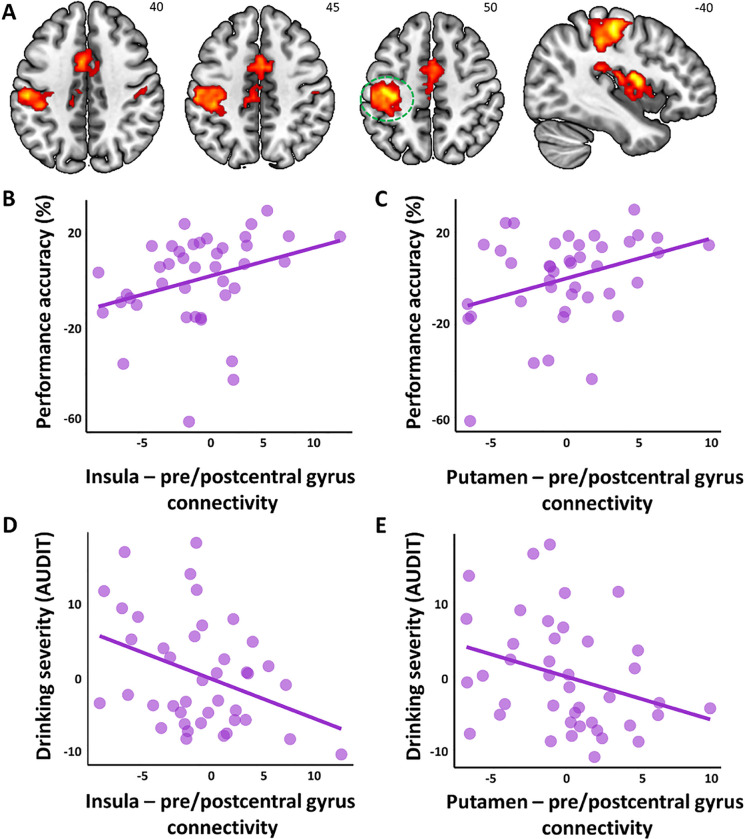
(**A**) Regions involved in proactive avoidance were identified in social drinkers: bilateral but primarily left pre/postcentral gyri, bilateral insula, left middle frontal gyrus, dorsal anterior cingulate cortex, supplementary motor area, left thalamus, and right cerebellum. We focused on the left pre/postcentral gyrus (in green circle) for subsequent functional connectivity analyses. In problem drinkers, proactive avoidance performance accuracy showed significant and positive correlations with (**B**) insula-pre/postcentral gyrus connectivity and (**C**) putamen-pre/postcentral gyrus connectivity. In contrast, drinking severity showed significant and negative correlations with (**D**) insula-pre/postcentral gyrus connectivity and (**E**) putamen-pre/postcentral gyrus connectivity.

To better understand the relationship between proactive avoidance deficits and the attenuation of the insular and putamen activities, we conducted an exploratory connectivity analysis. Using gPPI, we focused on the connectivity between the insula/putamen and brain regions involved during proactive avoidance in problem drinkers. To first identify the regions showing enhanced activation during proactive avoidance, we employed the contrast Go-to-avoid > Nogo-to-Avoid in social drinkers which showed significant activation in the bilateral but primarily left pre/postcentral gyri, bilateral insula, left middle frontal gyrus, dorsal anterior cingulate cortex, supplementary motor area, left thalamus, and right cerebellum (*Supplementary Fig. S4, Table S3*). We focused on the left pre/postcentral gyrus (Fig. 6A) as the region is implicated in higher-order motor processing during avoidance learning ^53,54^. Importantly, our own previous work has implicated the connectivity between the left precentral gyrus and the affective circuits in driving alcohol use ^52^.

Next, we conducted a gPPI analysis using the insula and putamen as seeds during proactive avoidance in problem drinkers. The parameter estimates were extracted for the connectivity strength between the right insula and left pre/postcentral gyrus as well as the right putamen and left pre/postcentral gyrus. Finally, we examined the relationship of these connectivities with proactive avoidance performance and drinking severity. We found that Go-to-avoid performance accuracy was significantly and positively correlated with the insula-pre/postcentral gyrus connectivity (*r* = .36, *p* = .033, Fig. 6B) we well as the putamen-pre/postcentral gyrus connectivity (*r* = .38, *p* = .023, Fig. 6C). Conversely, drinking severity was significantly and negatively correlated with the insula-pre/postcentral gyrus connectivity (*r* = − .39, *p* = .019, Fig. 6D) we well as the putamen-pre/postcentral gyrus connectivity (*r* = − .35, *p* = .039, Fig. 6E). Thus, reduced insular and putamen connectivity with the pre/postcentral gyrus was associated with decreased proactive avoidance performance and increased drinking severity in problem drinkers.

For completeness, we extracted the parameter estimates of the insular and putamen connectivity with other regions involved in proactive avoidance (i.e., middle frontal gyrus, dorsal anterior cingulate cortex, supplementary motor area, thalamus, and cerebellum). No connectivity showed significant relationships with drinking severity or proactive avoidance task performance (*p*’s > .10).

## Discussion

The current study examined the neural processes underlying impaired proactive avoidance and how these processes may be influenced by alcohol use severity and drinking motives in problem drinkers. Behaviorally, problem drinkers exhibited significantly lower learning rate and performance accuracy than social drinkers in proactive avoidance. These deficits were associated with higher alcohol use severity as well as stronger motives both to alleviate negative emotion and physical pain. In problem drinkers, attenuation of the right insula and right putamen activities during proactive avoidance predicted greater negative emotions and physical pain drinking motives, respectively. Insula and putamen activations as well as functional connectivity with the left pre/postcentral gyrus also exhibited a negative and positive relationship, respectively, with alcohol use severity and proactive avoidance task performance. Finally, we used path models to characterize how drinking motives and alcohol use severity may have weakened insular and putamen responses to proactive avoidance, thus compromising learning as well as behavioral performance. These findings highlight a potential neurobehavioral mechanism perpetuating problem drinking.

### Impaired proactive avoidance in problem drinking

Proactive avoidance mitigates drinking motivated by physical and emotional distress ^60,61^. In real life, proactive avoidance may involve actively solving problems, regulating emotions, gaining sense of control, and seeking support in response to potential aversive events. Studies have reported the positive effects of these proactive behaviors in lowering alcohol consumption in problem drinkers and decreasing the risks of relapse in those receiving treatment for AUD ^62–64^. Conversely, reduced proactive avoidance have been linked to greater alcohol use severity and relapse ^8–10,65^. More broadly, as an effective problem-focused coping strategy, proactive avoidance has been employed in the treatment of anxiety disorders ^66^. As anxiety can contribute to alcohol misuse ^67^, it may be beneficial to incorporate proactive avoidance in interventions against problem drinking.

The current study offered robust experimental evidence for compromised proactive avoidance – the initiation of an action to avoid painful outcomes – in problem drinkers. Importantly, this proactive avoidance deficit, manifested as lowered learning rate and performance accuracy, was associated with both greater alcohol use severity and drinking-to-cope motives. It is plausible that, along with being impaired in proactive avoidance, problem drinkers may be motivated to seek alcohol reactively or impulsively to mitigate physical pain and negative emotions. Due to poor learning, these individuals likely fail to benefit from adaptive coping strategies and instead rely on maladaptive behaviors. As such, proactive avoidance may represent both a predictor of drinking behavior and an intervention target for treatment of alcohol misuse.

### Insula’s and putamen’s roles in proactive avoidance and problem drinking

We found that heightened motive to lessen negative emotions through drinking was predictive of lower right insular activity during proactive avoidance in problem drinkers. The literature has extensively highlighted the role of the insula in processing negative emotions such as fear, disgust, unfairness, and social rejection ^68–71^. Insular hyperactivity in visceral interoception has also been associated with affective disorders ^72,73^, a frequent comorbidity of alcohol misuse. Notably, it was reported that alcohol-dependent individuals but not healthy controls showed greater right insular activations during exposure to negative vs. neutral images ^74^. A study employing the Cyberball paradigm found that people with AUD, relative to healthy controls, exhibited greater right insular activation to social ostracism ^75^. These findings may suggest a role of the right insula in coping with aversive emotions in problem drinkers. Additionally, the insula is implicated in promoting drug urges ^76,77^, alcohol cue-induced craving in heavy drinkers ^78^ as well as stress-related craving in those with AUD ^79^. These findings thus provide a potential link between negative emotional states and craving as well as seeking of alcohol.

Insula may have an additional role in the facilitation of avoidance behavior in response to negative emotions ^80,81^. Several investigations have reported that the right insula increased activation to cues predictive of punishments such as aversive tones ^82^, bitter taste ^83^, and financial loss ^84^. Thus, the region may contribute to actions aimed at avoiding negative consequences. Other works indeed implicated the insula in avoidance learning and in the initiation of behaviors to minimize future harms. For instance, using a monetary incentive delay task, it was found that insular response to punishment predicted participants’ ability to learn to avoid subsequent losses ^27^. In another study, the right insula’s activity following punishment was associated with higher probability of choosing safe responses in future trials ^85^. These reports are also in support of our path model finding that the weakening of the insula activation was associated with worse proactive avoidance learning rate. As such, the insula likely represents a neural correlate not only of the experience or anticipation of aversive outcomes but also of the integration of motivational signals in goal-directed avoidance ^54^. In alcohol use, our evidence of a four-way relationship between attenuated right insula activity, heightened negative emotions drinking motive, impaired proactive avoidance, and increased alcohol use severity shed light on the insula’s complex role in drinking behavior. Adding to a literature of the region’s involvement in the hedonic experience of drug use and drug urges ^86,87^, the present work points to the insula-centered multiple processes underlying the pathophysiology of problem drinking.

In contrast, the right putamen responded to proactive avoidance in negative association with physical pain drinking motive, suggesting that physical pain and negative emotion may drive alcohol use via at least partially distinct mechanisms. Past research has implicated the putamen in pain processing, as reported in a meta-analysis of brain activation to the sensation of physical pain in humans ^88^. In rodents, the putamen was found to contain cholinergic neurons that modulate nociceptive signals ^89^. Further, the putamen has a high density of opioid receptors ^90^. A positron emission tomography study of humans reported endogenous opioid releases in response to pain in the right putamen ^91^, indicating a role of the putamen in pain relief. In studies employing behavioral tasks of avoidance learning, the putamen showed higher activation to the initiation of an action to avoid electric shocks ^92^ and aversive visual stimuli ^93^. Additionally, individual harm avoidance trait was positively associated with putamen activity during risk-taking in the stop-signal task ^94^. In monkeys, tonically active neurons in the putamen were shown to respond to action initiation during learning to obtain reward and avoid aversive air puffs ^95^. Our own evidence of a positive relationship between putamen activation and proactive avoidance learning rate is thus in line with past reports on putamen’s involvement in avoidance behaviors. Taken together, the literature and our findings provide important evidence linking proactive avoidance in response to physical pain and the weakening of putamen activity during proactive avoidance in problem drinkers.

Our path analysis described two pathways through which drinking motives negatively affect proactive avoidance potentially via the effects of harmful alcohol use. In the first, negative emotions promote problem drinking which, in turn, reduces insular activity and leads to proactive avoidance learning and performance deficits. In the second, physical discomfort promotes problem drinking and leads to deficits in proactive avoidance via the attenuation of putamen involvement. Previous studies have associated alcohol misuse with negative physical and emotional motives ^43,96,97^ and demonstrated reduction in proactive behaviors in problem drinkers ^4,98,99^. Adding to this literature, the current findings are the first to elucidate the neural processes inter-linking alcohol use severity, drinking motives, and deficits in proactive avoidance.

### The role of somatomotor cortex in proactive avoidance and drinking

Our functional connectivity results showed reduced insular and putamen connectivity with the left pre/postcentral gyrus – the somatomotor cortex (SMC) – in association with higher drinking severity and lower proactive avoidance performance. These findings shed additional light on the negative impacts of insular and putamen disengagement, suggesting that harmful drinking may be associated with changes in brain processes inter-regionally. The SMC has been implicated in the initiation of avoidance behavior. For instance, neurons in the monkey’s precentral gyrus became active during defensive reactions against air puffs ^100^. The postcentral gyrus in non-human primates has also been found to encode nociceptive inputs, possibly helping to facilitate avoidance ^101^. In humans, studies have associated activation of the left pre and postcentral gyrus with avoidance in behavioral tasks that investigated risky decisions ^102^, avoidance of financial punishment ^103^, individual variation in loss aversion ^104^, and stimulus-triggered phobia ^105^. Thus, reduced insular and putamen connectivity may lead to impaired signaling of the SMC to initiate avoidance behavior. With regarding to drinking, the current work provided evidence for clinical implications of these dysfunctional circuits by associating them with proactive avoidance deficits and ultimately alcohol use severity. Along with our earlier report of left precentral gyrus connectivity with the periaqueductal gray and medial orbitofrontal cortex modulating alcohol use ^52^, the present findings provide broad support for a functional role of the SMC in alcohol misuse.

### Limitations and conclusions

The present study has several limitations. First, the sample size was moderate and there were significantly more males than females in the problem drinker group. While we controlled for sex in our analysis of the whole sample, a larger and more balanced sample would have allowed us to examine sex differences in these findings. Further, as the bulk of the literature focused on financial loss to study avoidance, we employed avoidance of physical pain (i.e., electric shocks) in the PLGT with the belief that this would most reliably elicit psychological and brain responses mimicking those experienced by drinkers in pain and negative emotional states. Thus, more studies are needed to assess how these differences may influence the results.

Individuals may resort to alcohol use to alleviate physical pain and negative emotions as a maladaptive behavior, thus perpetuating drinking. In contrast, proactive avoidance protects against problem drinking via the promotion of actions aimed at addressing and/or preventing negative events that could induce alcohol craving. We found that the right insula and putamen may relay signals of negative emotions and physical pain, respectively, to the cognitive motor circuits in supporting proactive avoidance. Likely due to the effects of chronic alcohol exposure, problem drinkers show deficits in association with attenuated insular and putamen during proactive avoidance. These findings offer the first experimental evidence of impaired proactive avoidance in problem drinkers with implications for behavioral treatment of alcohol use disorders.
